# Receipt of Combined Axillary Dissection and Nodal Irradiation Varies by Age

**DOI:** 10.1245/s10434-026-19746-3

**Published:** 2026-05-08

**Authors:** Sara P. Myers, Yevgeniya Gokun, Brandon Slover, shley P. Davenport, herese Y. Andraos, Nerea Lopetegui-Lia, Heather LeFebvre, Daniel G. Stover, Elizabeth A. Mittendorf, Tari A. King, Olga Kantor

**Affiliations:** 1 Division of Breast Surgery, Department of Surgery, The Ohio State University, Columbus, OH USA; 2 Center for Biostatistics, College of Medicine, The Ohio State University, Columbus, OH USA; 3 Division of Medical Oncology, Department of Internal Medicine, The Ohio State University Comprehensive Cancer Center, Columbus, OH USA; 4 Department of Radiation Oncology, The Ohio State University Comprehensive Cancer Center, Columbus, OH USA; 5 Division of Breast Surgery, Department of Surgery, Brigham and Women’s Hospital, Boston, MA USA; 6 Breast Oncology Program, Dana-Farber Cancer Institute, Boston, MA USA; 7 Harvard Medical School, Boston, MA USA

**Keywords:** Breast cancer, Axillary lymph node dissection, Regional nodal irradiation, Breast cancer-related lymphedema, Age

## Abstract

**Background:**

Combination axillary lymph node dissection (ALND) and regional nodal irradiation (RNI) poses the greatest risk for breast cancer-related lymphedema. Despite interest in minimizing combination therapy (i.e., ALND+RNI) to avoid complications, concern remains over the oncologic safety of de-escalation in younger populations. This retrospective analysis explores the association between age and receipt of ALND+RNI to gain insight into patterns of axillary management.

**Methods:**

Using the National Cancer Database (2018–2020), age-based differences in ALND+RNI among patients with stage I–III breast cancer undergoing surgery were examined. Patient and treatment characteristics were compared by age (<45, 45–64, and ≥65 years). Multivariable regression assessed associations between age and treatment, adjusting for clinical factors.

**Results:**

Among 439,790 patients, 7.5% were aged <45 years, 45.2% were 45–64, and 47.3% were ≥65. Patients aged <45 years (9.9%) were more commonly clinically node-positive (cN+) (vs 5.7% for age 45–64 and 5.5% for age ≥65 years; *p*<0.001). Younger patients were more likely to undergo ALND (23.8% vs 17.0% vs 16.3%), RNI (23.1% vs 16.9% vs 12.9%), and ALND+RNI (10.6% vs 6.2% vs 4.6%) (all *p*<0.001). On multivariable analysis, age <45 years had the highest odds of ALND+RNI (odds ratio 1.11; 95% confidence interval 1.03–1.20 vs ≥65 years) overall, but age did not impact ALND+RNI among those receiving neoadjuvant chemotherapy (*p*=0.209). On sensitivity analyses, those with cN+ molecular subtypes were more likely to receive ALND+RNI than those with clinical N0 disease.

**Conclusion:**

Women aged <45 years are more likely to receive ALND+RNI. These data highlight an opportunity for further studies targeting management in younger cohorts to limit overtreatment.

Breast cancer is the second most common malignancy among women and remains a major source of morbidity and mortality.^1^ Therapies have evolved significantly over past decades, with treatment strategies personalized based on tumor biology, stage, and patient-level factors.^2^ In particular, age-related differences in pathophysiology may impact treatment decisions and outcomes. Existing evidence has demonstrated that younger patients (i.e., aged ≤45 years at the time of diagnosis) are more likely to present with aggressive subtypes requiring multimodality treatment.^3^ In contrast, postmenopausal women commonly present with hormone-receptor-positive disease, a more favorable subtype^4^ for which de-escalation of therapy may be appropriate.^5^

Consistent with these efforts toward de-escalation, clinical guidelines have increasingly emphasized de-implementation of ‘low-value,’ axillary surgery that may provide little benefit but potentially cause harm. With the introduction of sentinel lymph node biopsy (SLNB) as an accurate and less invasive approach for axillary staging, seminal studies have demonstrated the safety of omitting axillary lymph node dissection (ALND) in select populations. The American College of Surgeons Oncology Group Z0011 (ACOSOG) phase III randomized clinical trial reported long-term follow-up data that demonstrated that omission of ALND (i.e., SLNB alone) was noninferior to ALND in terms of overall survival for patients with early-stage clinically node-negative primary breast cancer undergoing planned breast conservation surgery.^6^ Subsequently, multiple other studies have substantiated omission of ALND and helped refine criteria for appropriate candidates.^7–11^ This paradigm shift reduces treatment-related morbidity resulting from therapies that do not meaningfully improve oncologic outcomes. Specifically, axillary surgery and radiation increase the risk of breast cancer-related lymphedema (BCRL), a common and debilitating lifelong ailment that occurs in more than 20% of breast cancer survivors^12^^,^^13^ and has been shown to have profound consequences on quality of life.^14^^,^^15^

Despite efforts to avoid ALND, data in support of this practice may not be generalizable to young adults as this population was underrepresented in seminal studies investigating the safety of de-escalating axillary surgery. Additionally, given the proportion of aggressive tumor subtypes, a higher proportion of young adults may receive neoadjuvant systemic therapy. Although data from the Alliance A011202 trial^16^ will inform practice standards for axillary surgery in this context, data on managing residual disease after preoperative treatment are nascent. Given the prevalence of multimodality treatment among young adult patients with breast cancer, further research is essential to understand how age impacts treatment decision-making, an important step toward identifying risk factors for treatment-related adverse events. In this study, we examine age-related differences in the use of comprehensive axillary management with ALND and regional nodal irradiation (RNI) using data from the National Cancer Database (NCDB), hypothesizing that patients aged ≤45 years are more likely to undergo combination therapy (i.e., ALND+RNI) even after accounting for clinicopathologic characteristics. Insights into treatment patterns may offer opportunities for further studies to support practice changes that prevent overtreatment and reduce treatment-related adverse events, including BCRL.^17^

## Methods

### Study Design and Population

**Fig. 1:**
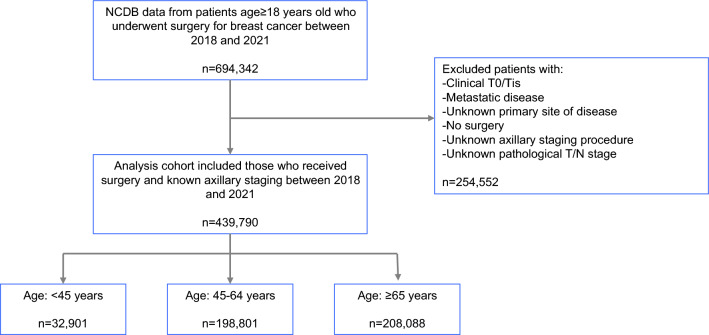
Study schema. NCDB, National Cancer Database

In this retrospective study, the NCDB was used to identify adult patients aged ≥18 years with stage I–III breast cancer who underwent definitive surgery between 2018 and 2021. The year 2018 was selected as the starting date for inclusion because data related to RNI fields were not clearly defined in the NCDB before this. Patients with unknown axillary staging procedure, pathological T stage, or pathological N stage were excluded (Fig. 1).

### Sociodemographic and Clinical Characteristics

The primary exposure of interest was age at breast cancer diagnosis, categorized as follows: aged <45 years, 45–64 years, and ≥65 years. A threshold of 45 years was chosen to define younger age, reflecting epidemiologic trends of rising early-onset cancer rates that extend beyond the traditional 18–39-year range associated with young adult cancers.^18^ The primary outcome was the receipt of comprehensive axillary management with ALND+RNI. Those who received both were coded as ‘yes’, and the receipt of neither or only one of two were coded as ‘no’. Other clinically relevant covariates that were considered included sociodemographic factors: race, ethnicity, household income, insurance status, educational attainment, residing in a rural versus urban area, and distance traveled to treatment facility. Household income at diagnosis was classified by median quartile based on income ranges for US zip code as determined by the US Census.^19^ Insurance status was categorized as not insured, private insurance/managed care, Medicaid, Medicare, or other government. Percentage of adults with less than high school education was based on zip code, and this educational attainment for the patient’s area of residence was included as a surrogate for social mobility.^20^ The final dataset also incorporated clinical, disease, and treatment characteristics, including Charlson–Deyo comorbidity scale, tumor histology, clinical and pathological T and N stages, molecular subtype, systemic chemotherapy (neoadjuvant vs adjuvant), surgery (breast and axilla), and endocrine therapy. Molecular subtype was defined based on hormone receptor (HR; estrogen and/or progesterone receptor) and human epidermal growth factor receptor 2 (HER2) status as follows: hormone receptor positive, HER2 negative (HR+/HER2−), HR+/HER2+, HR−/HER2+, and HR-/HER2-.^21^ Surgical procedures were further classified as breast surgery (lumpectomy vs mastectomy), reconstruction (binary yes vs no), and axillary management procedure (no axillary surgery, SLNB, or ALND). Adjuvant radiation was classified as no radiation, chest wall/breast only, and chest wall/breast+RNI.

### Statistical Analysis

Patient, tumor, and treatment characteristics were compared among women between the three age categories using descriptive statistics with frequencies and percentages for categorical variables. Differences between these groups were calculated using chi-squared tests. Multivariable logistic regressions were performed to evaluate the association between age and receipt of comprehensive axillary management while adjusting for sociodemographic and clinical variables. Multivariable regressions were also stratified by receipt of neoadjuvant versus adjuvant systemic therapy to determine whether associations between age and ALND+RNI differed based on sequence of treatment. Additionally, sensitivity analyses were performed to determine whether the association between tumor molecular subtype and likelihood of receiving comprehensive axillary management differed significantly by clinical nodal status adjusting for all the other variables specified in the main model. All analyses were performed using the SAS software (version 9.4; SAS Institute, Cary, NC, USA). A two-sided p-value <0.05 was considered statistically significant.

## Results

Of 439,790 patients, 7.5% were aged <45 years, 45.2% were 45–64 years, and 47.3% were ≥65 years (Table 1). Clinically node-positive disease was more common in those aged <45 years (9.9%) than among those aged 45–64 years (5.7%) or ≥65 years (5.5%) (*p*<0.001). Similarly, 31.9% of patients aged <45 years had pathologic node-positive disease versus 21.8% of those aged 45–64 years and 18.2% of those aged ≥65 years (*p*<0.001). Among all patients, 0.8% had no axillary staging, 82.0% had SLNB alone, and 17.2% had ALND. The majority of ALND occurred in patients who underwent upfront surgery, with 4.9% (1214/27,085) occurring after neoadjuvant systemic therapy. Patients aged <45 years were more likely to undergo ALND (23.8% vs 17.0% and 16.3% in age 45–64 years and ≥65 years, respectively, *p*<0.001). RNI (23.1% vs 16.9% and 12.9% in age 45–64 years and ≥65 years, respectively, *p*<0.001) and both ALND and RNI (10.6% vs 6.2% and 4.6% in age 45–64 years and ≥65 years, respectively, *p*<0.001).

**Table 1:** Patient, tumor, and treatment characteristics by age

Characteristics	Total (n=439,790)	<45 years (n=32,901)	45–64 years (n=198,801)	≥65 years (n=208,088)	P-value
Race					<0.001
White	367,017 (84.2)	24,898 (76.5)	162,305 (82.4)	17,9814 (87.0)	
African American/Black	43,111 (9.9)	4123 (12.7)	20,944 (10.6)	18,044 (8.7)	
Asian/Pacific Islander	19,148 (4.4)	2639 (8.1)	10,083 (5.1)	6426 (3.1)	
Other	6771 (1.6)	872 (2.7)	3581 (1.8)	2318 (1.1)	
Hispanic ethnicity	26,003 (6.0)	3516 (10.9)	13,882 (7.1)	8605 (4.2)	<0.001
Less than high school education					<0.001
≥15.3%	60,422 (13.7)	4937 (15.0)	27,788 (14.0)	27,697 (13.3)	
9.1–15.2%	95,304 (21.7)	7021 (21.3)	42,455 (21.4)	45,828 (22.0)	
5.0–9.0%	113,077 (25.7)	8273 (25.1)	50,848 (25.6)	53,956 (25.9)	
<5.0%	98,833 (22.5)	7740 (23.5)	45,751 (23.0)	45,342 (21.8)	
Unknown	72,154 (16.4)	4930 (15.0)	31,959 (16.1)	35,265 (16.9)	
Median income quartiles, $US					<0.001
<$46,227	47,550 (10.8)	3268 (9.9)	20,860 (10.5)	23,422 (11.3)	
$46,227–57,856	72,294 (16.4)	4921 (15.0)	31,388 (15.8)	35,985 (17.3)	
$57,587–74,062	87,964 (20.0)	6342 (19.3)	38,581 (19.4)	43,041 (20.7)	
≥$74,063	159,051 (36.2)	13,397 (40.7)	75,711 (38.1)	69,943 (33.6)	
Unknown	72,931 (16.6)	4973 (15.1)	32,261 (16.2)	35,697 (17.2)	
Insurance					<0.001
No insurance	4509 (1.0)	721 (2.2)	3115 (1.6)	673 (0.3)	
Managed care/private	208,646 (47.9)	26,400 (81.1)	156,800 (79.6)	25,446 (12.3)	
Medicaid	24,503 (5.6)	4099 (12.6)	17,557 (8.9)	2847 (1.4)	
Medicare	193,293 (44.3)	753 (2.3)	16,129 (8.2)	176,411 (85.5)	
Other government	5020 (1.2)	582 (1.8)	3374 (1.7)	1064 (0.5)	
Histology category					<0.001
Invasive ductal carcinoma	345,502 (78.6)	27,977 (85.0)	158,539 (79.7)	158,986 (76.4)	
Invasive lobular carcinoma	54,274 (12.3)	2335 (7.1)	23,305 (11.7)	28,634 (13.8)	
Mixed lobular + ductal carcinoma	18,751 (4.3)	1236 (3.8)	8713 (4.4)	8802 (4.2)	
Other	21,263 (4.8)	1353 (4.1)	8244 (4.1)	11,666 (5.6)	
Subtype					<0.001
ER/PR+, HER2+	19,999 (4.5)	2262 (6.9)	9304 (4.7)	8433 (4.1)	
ER/PR+, HER2-	330,515 (75.2)	25,436 (77.3)	151,619 (76.3)	153,460 (73.7)	
ER/PR-, HER2+	7057 (1.6)	557 (1.7)	3340 (1.7)	3160 (1.5)	
ER/PR-, HER2-	28,291 (6.4)	2020 (6.1)	11,369 (5.7)	14,902 (7.2)	
Unknown	53,928 (12.3)	2626 (8.0)	23,169 (11.7)	28,133 (13.5)	
Breast surgery type					<0.001
Breast conservation therapy	301,487 (68.6)	14,116 (42.9)	133,667 (67.3)	153,704 (73.9)	
Mastectomy	138,186 (31.4)	18,771 (57.1)	65,077 (32.7)	54,338 (26.1)	
Endocrine therapy receipt	352,599 (81.5)	26,877 (83.3)	163,834 (83.8)	161,888 (79.0)	<0.001
Axillary staging procedure					<0.001
No axillary surgery	3542 (0.8)	117 (0.4)	791 (0.4)	2634 (1.3)	
SLNB	360,774 (82.0)	24,967 (75.9)	164,256 (82.6)	171,551 (82.4)	
ALND	75,474 (17.2)	7817 (23.8)	33,754 (17.0)	33,903 (16.3)	
RNI	67,977 (15.5)	7605 (23.1)	33,557 (16.9)	26,815 (12.9)	<0.001
RNI and ALND	25,215 (5.7)	3490 (10.6)	12,253 (6.2)	9472 (4.6)	<0.001
Chemotherapy receipt					<0.001
None	324,563 (78.3)	16,176 (55.4)	138,310 (74.5)	170,077 (85.1)	
Neoadjuvant chemotherapy	6612 (1.6)	1292 (4.4)	3564 (1.9)	1756 (0.9)	
Adjuvant chemotherapy	83,516 (20.1)	11,720 (40.2)	43,813 (23.6)	27,983 (14.0)	
Radiation combo					<0.001
None	145,526 (33.3)	13,339 (40.9)	55,346 (28.0)	76,841 (37.2)	
Chest wall or breast only	223,630 (51.2)	11,717 (35.9)	108,698 (55.1)	103,215 (49.9)	
Chest wall or breast AND RNI	67,721 (15.5)	7577 (23.2)	33,418 (16.9)	26,726 (12.9)	
Clinical T stage					<0.001
T1	337,765 (76.9)	21,263 (64.7)	154,149 (77.6)	162,353 (78.1)	
T2	89,912 (20.5)	10,095 (30.7)	39,475 (19.9)	40,342 (19.4)	
T3	9640 (2.2)	1381 (4.2)	4289 (2.2)	3970 (1.9)	
T4	2005 (0.5)	113 (0.3)	656 (0.3)	1236 (0.6)	
Clinical N stage					<0.001
N0	412,848 (94.0)	29,566 (90.1)	186,921 (94.2)	196,361 (94.5)	
N1	24,080 (5.5)	2946 (9.0)	10,614 (5.3)	10,520 (5.1)	
N2	1477 (0.3)	210 (0.6)	660 (0.3)	607 (0.3)	
N3	583 (0.1)	101 (0.3)	263 (0.1)	219 (0.1)	
Pathological T stage					<0.001
T0	2269 (0.5)	402 (1.2)	1165 (0.6)	702 (0.3)	
T1	314,420 (71.5)	19,745 (60.0)	144,366 (72.6)	150,309 (72.2)	
T2	106,873 (24.3)	10,922 (33.2)	46,590 (23.4)	49,361 (23.7)	
T3	14,016 (3.2)	1730 (5.3)	6071 (3.1)	6215 (3.0)	
T4	2212 (0.5)	102 (0.3)	609 (0.3)	1501 (0.7)	
Pathological N stage					<0.001
N0	347,909 (79.1)	22,405 (68.1)	155,475 (78.2)	170,029 (81.7)	
N1	57,469 (13.1)	6496 (19.7)	27,215 (13.7)	23,758 (11.4)	
N1mi	18,053 (4.1)	2082 (6.3)	8810 (4.4)	7161 (3.4)	
N2	11,395 (2.6)	1404 (4.3)	5120 (2.6)	4871 (2.3)	
N3	4964 (1.1)	514 (1.6)	2181 (1.1)	2269 (1.1)	
Charlson–Deyo Score					<0.001
0	357,950 (81.4)	30,299 (92.1)	168,927 (85.0)	158,724 (76.3)	
1	56,045 (12.7)	2201 (6.7)	22,279 (11.2)	31,565 (15.2)	
2	15,424 (3.5)	291 (0.9)	4919 (2.5)	10,214 (4.9)	
3+	10,371 (2.4)	110 (0.3)	2676 (1.3)	7585 (3.6)	
Distance from hospital, miles					<0.001
<25	300,136 (68.2)	22,600 (68.7)	135,286 (68.1)	142,250 (68.4)	
25–50	42,882 (9.8)	3196 (9.7)	20,155 (10.1)	19,531 (9.4)	
>50	27,649 (6.3)	2327 (7.1)	12,734 (6.4)	12,588 (6.0)	
Unknown	69,123 (15.7)	4778 (14.5)	30,626 (15.4)	33,719 (16.2)	
Reconstruction	47,347 (10.8)	9841 (29.9)	28,359 (14.3)	9147 (4.4)	<0.001

Data are presented as n (%) unless otherwise indicated.

ALND, axillary lymph node dissection; ER, estrogen receptor; HER2, human epidermal growth factor receptor 2; PR, progesterone receptor; RNI, regional nodal irradiation; SLNB, sentinel lymph node biopsy

Of the 25,215 patients who received comprehensive axillary management, 2493 (9.9%) had pN0 and 822 (3.3%) had pN1mi disease (Table 2). Comprehensive axillary management among patients who were cN0, underwent upfront surgery, and had pN0/pN1mi disease was less common in women aged <45 years old than in those in older age groups (376/3490 [10.8%] <45 years vs 1646/12253 [13.4%] 45–64 years vs 1293/9472 [13.7%] ≥65 years; *p*<0.001). However, irrespective of RNI, ALND itself was more common in younger patients with cN0 undergoing upfront surgery with pN0, pNmi, or pN1 with one to two positive lymph nodes (3657/24,563 [14.9%] <45 years vs 19,085/168,172 [11.3%] 45–64 years vs 20,663/183,598 [11.3%] >65 years; *p*<0.001) (Table 3).

**Table 2:** Patient, tumor, and treatment characteristics for patients receiving combined axillary lymph node dissection and regional nodal irradiation

Characteristics	Total (n=25,215)
Age, years	
<45	3490 (13.8)
45–64	12,253 (48.6)
≥65	9472 (37.6)
Race	
White	20,208 (80.9)
African American/Black	3117 (12.5)
Asian/Pacific Islander	1207 (4.8)
Other	452 (1.8)
Ethnicity	
Non-Hispanic	22,776 (92.0)
Hispanic	1979 (8.0)
Less than high school education	
≥15.3%	3878 (15.4)
9.1–15.2%	5737 (22.8)
5.0–9.0%	6284 (24.9)
<5.0%	5153 (20.4)
Unknown	4163 (16.5)
Median income quartiles, $US	
<$46,227	3085 (12.2)
$46,227–57,856	4424 (17.5)
$57,587–74,062	5143 (20.4)
≥$74,063	8352 (33.1)
Unknown	4211 (16.7)
Insurance	
No insurance	452 (1.8)
Managed care/private	13,126 (52.5)
Medicaid	2056 (8.2)
Medicare	9020 (36.1)
Other government	339 (1.4)
Histology category	
Invasive ductal carcinoma	18,607 (73.8)
Invasive lobular carcinoma	4471 (17.7)
Mixed lobular + ductal carcinoma	1406 (5.6)
Other	731 (2.9)
Subtype	
ER/PR+, HER2+	1173 (4.7)
ER/PR+, HER2-	19,175 (76.0)
ER/PR-, HER2+	405 (1.6)
ER/PR-, HER2-	1462 (5.8)
Unknown	3000 (11.9)
Breast surgery type	
Breast conservation therapy	9472 (37.6)
Mastectomy	15,725 (62.4)
Endocrine therapy receipt	
Yes	21,986 (88.2)
No	2946 (11.8)
Chemotherapy receipt	
None	8459 (36.7)
Neoadjuvant chemotherapy	1515 (6.6)
Adjuvant chemotherapy	13,067 (56.7)
Clinical T stage	
T1	11,090 (44.1)
T2	10,884 (43.2)
T3	2640 (10.5)
T4	553 (2.2)
Clinical N stage	
N0	14,261 (56.7)
N1	9959 (39.6)
N2	657 (2.6)
N3	289 (1.1)
Pathological T stage	
T0	235 (0.9)
T1	8503 (33.7)
T2	11,727 (46.5)
T3	4161 (16.5)
T4	589 (2.3)
Pathological N stage	
N0	2493 (9.9)
N1	12,412 (49.2)
N1mi	822 (3.3)
N2	6449 (25.6)
N3	3039 (12.1)
Charlson–Deyo score	
0	20,424 (81.0)
1	3291 (13.1)
2	865 (3.4)
3+	635 (2.5)
Distance from hospital, miles	
<25	16,949 (67.2)
25–50	2573 (10.2)
>50	1685 (6.7)
Unknown	4008 (15.9)
Reconstruction	
Yes	4935 (19.6)
No	20,280 (80.4)

Data are presented as n (%).

ER, estrogen receptor; HER2, human epidermal growth factor receptor 2; PR, progesterone receptor

**Table 3:** Axillary staging procedures among patients with clinically node negative disease who underwent upfront surgery by age and pathologic nodal involvement

	Total			Age <45 years			Age 45–64 years			Age ≥65 years		
	pN0/pN1mi/ pN1 with 1–2 nodes n=376,333)	All other pN+ n=10,132)	P-value	pN0/pN1mi/ pN1 with 1–2 nodes (n=24,563)	All other pN+ (n=1156)	P-value	pN0/pN1mi/ pN1 with 1–2 nodes (n=168,172)	All other pN+(n=4782)	P-value	pN0/pN1mi/pN1 with 1–2 nodes (n=183,598)	All other pN+ (n=4194)	P-value
Axillary procedure			<0.001			<0.001			<0.001			<0.001
None	3199 (0.9)	21 (0.2)		71 (0.3)	3 (0.3)		633 (0.4)	9 (0.2)		2495 (1.4)	9 (0.2)	
SLNB	329,729 (87.6)	2191 (21.6)		20,835 (84.8)	208 (18.0)		148,454 (88.3)	1012 (21.2)		160,440 (87.4)	971 (23.2)	
Total ALND	43,405 (11.5)	7920 (78.2)		3657 (14.9)	945 (81.7)		19,085 (11.3)	3761 (78.6)		20,663 (11.3)	3214 (76.6)	
ALND	11,386 (3.0)	1574 (15.5)		700 (2.8)	146 (12.6)		4382 (2.6)	657 (13.7)		6304 (3.4)	771 (18.4)	
SLNB+ALND	32,019 (8.5)	6346 (62.6)		2957 (12.0)	799 (69.1)		14,703 (8.7)	3104 (64.9)		14,359 (7.8)	2443 (58.2)	

Data are presented as n (%) unless otherwise indicated.

ALND, axillary lymph node dissection; pN0, pathologically node negative; pN1, pathologically node positive with fewer than four lymph nodes involved; pN1mi, pathologically node positive with micrometastatic disease; SLNB, sentinel lymph node biopsy.

On multivariable analysis adjusting for factors determined to be clinically relevant *a priori*, age was inversely associated with receipt of ALND and RNI; patients aged <45 years had higher odds of receiving ALND and RNI (<45 vs ≥65 years: odds ratio [OR] 1.11; 95% confidence interval [CI] 1.03–1.20) (Table 4). Pathologic N category was associated with the greatest odds of undergoing ALND and RNI (pN1: OR 22.59; 95% CI 21.46–23.79; pN2: OR 64.94; 95% CI 60.72–69.46; pN3: OR 65.78; 95% CI 60.42–71.63 vs pN0). Hispanic ethnicity, lobular histology, higher clinical and pathologic T stages, clinical node positivity, mastectomy, and chemotherapy receipt were associated with higher odds of receiving ALND and RNI (Table 4). Factors associated with decreased odds of undergoing ALND with RNI included Medicare status and non-HR+/HER2- molecular subtypes. Race, proportion of high-school graduates in the patient’s residential area, and distance from the health center were not significantly associated with the receipt of comprehensive axillary management. In analyses stratified by timing of systemic therapy, younger age was associated with ALND+RNI among those receiving adjuvant chemotherapy (<45 vs ≥65 years: OR 1.16; 95% CI 1.05–1.29, *p*=0.014; Table 4). No age-based differences in ALND+RNI were noted among those receiving neoadjuvant chemotherapy (*p*=0.209).

**Table 4:** Multivariable analysis of variables associated with comprehensive axillary management

	All patients			Neoadjuvant			Adjuvant		
Variable	OR	95% CI	P-value	OR	95% CI	P-value	OR	95% CI	P-value
Age (years)			0.019			0.209			0.014
<45	1.11	1.03–1.20		1.07	0.81–1.41		1.16	1.05–1.29	
45–64	1.06	1.00–1.12		1.19	0.94–1.51		1.08	0.99–1.17	
≥65	Ref	Ref		Ref	Ref		Ref	Ref	
Race			0.553			0.411			0.556
Black	1.04	0.98–1.09		0.87	0.72–1.05		1.03	0.96–1.12	
Asian/Pacific Islander	1.02	0.94–1.11		0.92	0.67–1.26		1.04	0.93–1.16	
Other	1.04	0.91–1.18		0.82	0.53–1.27		1.10	0.91–1.33	
White	Ref	Ref		Ref	Ref		Ref	Ref	
Hispanic ethnicity	1.11	1.04–1.19	0.003	0.95	0.74–1.23	0.711	1.10	1.00–1.20	0.061
Less than high school education			0.326			0.212			0.146
≥15.3%	0.95	0.89–1.01		0.77	0.61–0.98		0.90	0.83–0.98	
9.1–15.2%	1.00	0.95–1.05		0.86	0.70–1.07		0.93	0.86–1.00	
5.0–9.0%	0.97	0.93–1.02		0.84	0.68–1.03		0.95	0.88–1.02	
Unknown	0.93	0.76–1.15		1.20	0.52–2.76		0.90	0.67–1.22	
<5.0%	Ref	Ref		Ref	Ref		Ref	Ref	
Insurance			0.020			0.068			0.193
No insurance	1.05	0.91–1.20		1.16	0.77–1.74		0.91	0.75–1.10	
Medicaid	0.95	0.89–1.01		0.79	0.63–1.00		0.97	0.88–1.06	
Medicare	0.92	0.87–0.97		0.99	0.78–1.26		0.91	0.83–0.99	
Other government	1.00	0.86–1.15		0.54	0.30–0.96		1.02	0.84–1.25	
Private	Ref	Ref		Ref	Ref		Ref	Ref	
Charlson–Deyo Comorbidity			0.025			0.240			0.055
1	1.05	1.00–1.10		0.81	0.64–1.03		1.05	0.98–1.13	
2+	0.94	0.88–1.01		0.95	0.68–1.33		0.90	0.81–1.01	
0	Ref	Ref		Ref	Ref		Ref	Ref	
Histology			<0.001			0.037			0.287
Invasive lobular carcinoma	1.14	1.08–1.19		1.43	1.12–1.82		1.07	0.99–1.15	
Mixed	1.02	0.95–1.10		1.04	0.73–1.49		0.98	0.88–1.10	
Other	0.99	0.89–1.09		0.98	0.66–1.43		1.06	0.91–1.23	
Invasive ductal carcinoma	Ref	Ref		Ref	Ref		Ref	Ref	
Tumor molecular subtype			<0.001			0.002			<0.001
ER/PR+/HER2+	0.59	0.52–0.67		0.67	0.45–0.99		0.52	0.43–0.64	
ER/PR-/HER2+	0.71	0.63–0.81		1.05	0.78–1.42		0.72	0.61–0.85	
ER/PR-/HER2-	0.77	0.72–0.83		0.71	0.59–0.86		0.84	0.77–0.92	
Unknown	0.88	0.83–0.92		0.88	0.72–1.07		0.85	0.79–0.91	
ER/PR+/HER2-	Ref	Ref		Ref	Ref		Ref	Ref	
Mastectomy	1.39	1.34–1.44	<0.001	1.18	1.01–1.38	0.038	1.57	1.49–1.66	<0.001
Clinical T stage			<0.001			0.004			0.003
T1	Ref	Ref		Ref	Ref		Ref	Ref	
T2	1.12	1.07–1.17		1.27	1.06–1.51		1.08	1.02–1.15	
T3/T4	1.13	1.05–1.22		1.43	1.15–1.77		1.17	1.06–1.31	
Clinical N+	2.30	2.21–2.39	<0.001	2.80	2.39–3.29	<0.001	1.98	1.88–2.10	<0.001
Pathologic T stage			<0.001			<0.001			<0.001
T0	4.09	3.26–5.13		1.61	1.24–2.10		1.74	0.38–8.02	
T1	Ref	Ref		Ref	Ref		Ref	Ref	
T2	1.13	1.08–1.18		1.09	0.92–1.30		1.14	1.07–1.21	
T3	1.46	1.36–1.56		1.40	1.09–1.79		1.35	1.23–1.48	
T4	0.89	0.78–1.02		0.76	0.51–1.14		1.16	0.95–1.41	
Pathologic N stage			<0.001			<0.001			<0.001
N0	Ref	Ref		Ref	Ref		Ref	Ref	
N1	22.59	21.46–23.79		4.46	3.64–5.47		24.31	22.02–26.83	
N1mi	5.03	4.61–5.49		3.21	2.36–4.39		5.12	4.37–6.00	
N2	64.94	60.72–69.46		9.39	7.39–11.93		86.70	77.70–96.74	
N3	65.78	60.42–71.63		10.24	7.54–13.90		93.10	81.93–105.79	
Distance from health center, miles			0.491			0.744			0.851
<25	Ref	Ref		Ref	Ref		Ref	Ref	
25–50	0.98	0.93–1.04		0.95	0.75–1.21		1.00	0.93–1.09	
>50	0.96	0.90–1.03		1.09	0.82–1.43		0.97	0.88–1.07	
Unknown	1.09	0.88–1.34		0.72	0.31–1.65		1.08	0.80–1.47	
Chemotherapy			<0.001						
Neoadjuvant	2.07	1.90–2.25							
Adjuvant	2.02	1.95–2.10							
None	Ref	Ref							

CI, confidence interval; ER, estrogen receptor; HER2, human epidermal growth factor receptor 2; OR, odds ratio; PR, progesterone receptor

Sensitivity analysis demonstrated that the association between tumor molecular subtype and likelihood of receiving ALND with RNI differed by clinical nodal status at presentation (interaction *p*<0.001; Table 5). All subtypes with clinically node-positive disease were significantly more likely to receive comprehensive axillary management than those with cN0 disease. Among those with cN0 disease, patients with HR+/HER2- tumors had the greatest odds of having ALND with RNI (Table 5).

**Table 5:** Sensitivity analysis exploring how association between clinical nodal status at presentation and comprehensive axillary management differs by tumor molecular subtype

**Predictor variable**	**OR**_**adj**_	**95% CI**	**P-value**
**Interaction: Subtype x clinical node positivity**			<0.001^a^
Among patients with subtype as ER/PR+/HER2+			0.002
Clinical N0	Ref		
Clinical N+	1.51	1.17–1.95	
Among patients with subtype as ER/PR+/HER2-			<0.001
Clinical N0	Ref		
Clinical N+	2.38	2.28–2.49	
Among patients with subtype as ER/PR-/HER2+			<0.001
Clinical N0	Ref		
Clinical N+	1.95	1.50–2.52	
Among patients with subtype as ER/PR-/HER2-			<0.001
Clinical N0	Ref		
Clinical N+	1.90	1.65–2.18	
Among patients with subtype as unknown			<0.001
Clinical N0	Ref		
Clinical N+	2.21	1.99–2.45	
Among patients with clinical N0			
ER/PR+/HER2+	0.71	0.60–0.83	<0.001
ER/PR-/HER2+	0.76	0.65–0.90	0.001
ER/PR-/HER2-	0.84	0.77–0.92	<0.001
Unknown	0.90	0.84–0.96	0.001
ER/PR+/HER2-	Ref		
Among patients with clinical N+			
ER/PR+/HER2+	0.45	0.36–0.55	<0.001
ER/PR-/HER2+	0.63	0.51–0.77	<0.001
ER/PR-/HER2-	0.67	0.60–0.75	<0.001
Unknown	0.83	0.76–0.91	<0.001
ER/PR+/HER2-	Ref		

OR_adj_ is adjusted OR for the following variables: age, race, ethnicity, education, insurance, Charlson–Deyo score, histology type, breast surgery, chemotherapy, clinical T stage, pathological T stage and pathological N stage.

95% CI is the corresponding 95% CI for the adjusted OR.

^a^ p-value is from a likelihood ratio chi-squared test for the interaction effect.

CI, confidence interval; ER, estrogen receptor; HER2, human epidermal growth factor receptor 2; OR_adj_, adjusted odds ratio; PR, progesterone receptor.

## Discussion

This retrospective analysis of women with stage I–III breast cancer treated between 2018 and 2021 reveals age-based differences in axillary disease burden and management strategies, with potentially important implications for treatment-related morbidity and long-term outcomes. Patients aged <45 years at diagnosis were more likely to have clinically and pathologically evident nodal disease than were older cohorts, and younger age was independently associated with higher odds of receiving comprehensive axillary management even after adjusting for tumor and treatment characteristics. Among those who received chemotherapy, younger age remained an important factor in comprehensive axillary management for those receiving adjuvant but not neoadjuvant systemic therapy. These data indicate that age may be an important consideration in assessing risk for overtreatment.

This study corroborates the work of others highlighting that younger age at diagnosis is associated with unfavorable histopathologic disease characteristics.^22^^,^^23^ Although early-onset breast cancer has been associated with a higher risk of recurrence and worse survival than an average age at onset,^24^^,^^25^ the likelihood of pathologic complete response (pCR) after preoperative systemic therapy may be greater in this demographic.^26^ In their analysis of age-based differences in pCR rates among 1383 patients with stage I–III breast cancer treated with neoadjuvant chemotherapy at Memorial Sloan Kettering Cancer Center between 2013 and 2018, Verdial et al.^27^ observed that, although overall pCR did not differ by age, there were differences among those with triple-negative disease; 52% of women aged <40 years with triple-negative tumors had experienced a pCR compared with 35% of those aged 41–60 years and 29% of those aged ≥61 years at the time of diagnosis. Moreover, among women with clinically node-positive disease, a significantly higher proportion of patients aged <40 years (52%) were able to avoid ALND after neoadjuvant therapy compared with older cohorts (39% of women aged 41–60 years, and 37% of women aged ≥61 years, *p*<0.001). This may be consistent with our findings that the odds of receiving ALND+RNI were not increased among younger patients receiving neoadjuvant systemic therapy. These data, taken with recently published findings from the NSABP B-51-Radiation Therapy Oncology Group 1304 trial that evaluated the impact of omitting RNI among patients with clinically node-positive breast cancer who experienced axillary nodal pCR, highlight opportunities to de-escalate treatments that do not confer added benefit. Such efforts to de-escalate may mitigate the risk of adverse events that could accompany these treatments (e.g., BCRL).

Just as in the neoadjuvant setting, opportunities to de-escalate axillary management also exist in the upfront surgery setting. As previously mentioned, ALND is now routinely omitted in clinically node-negative patients who have one or two positive axillary lymph nodes after breast conservation surgery and SLNB. Although omission of ALND among those who undergo mastectomy had been more controversial given the inclusion criteria for ACOSOG Z11,^6^ the AMAROS (10981-22023 Comparison of Complete Axillary Lymph Node Dissection with Axillary Radiation Therapy in Treating Women with Invasive Breast Cancer)^7^ and SENOMAC (Sentinel Node Biopsy in Breast Cancer: Omission of Axillary Clearance After Macrometastases)^10^ trials demonstrated the safety of de-implementing ALND for patients after upfront surgery with mastectomy and low-volume nodal burden. Despite these studies, practice has been slow to adapt. A recent study by Wang et al.^28^ using NCDB data from 2012 to 2021 showed that ~20% of patients who underwent mastectomy and axillary staging for clinically T1–2 node-negative disease and had fewer than three positive lymph nodes received post-mastectomy radiation with ALND. In our analysis of a more modern cohort of patients, approximately 15% of patients who had ALND could have been considered for omission based on evidence from previously mentioned studies. Furthermore, potential overtreatment with ALND differed by age and was more common among patients aged <45 years. Given that significant morbidity may result from overtreatment of the axilla, much of which may be delayed and disproportionately affect younger individuals with longer life expectancies, greater emphasis should be placed on eliminating unnecessary procedures and their concomitant risk.

The associations between histopathologic features and the likelihood of receiving comprehensive axillary management that were observed in this study are consistent with existing evidence. In this study, compared with ductal histology, patients with lobular carcinoma had higher odds of undergoing ALND with RNI. These data can be contextualized within a precision oncology framework that leverages tailored preoperative systemic therapy to downstage disease.^29^ Although lobular cancers are characterized by a diffuse growth pattern that obfuscates early diagnosis,^30^^,^^31^ higher nodal disease burdens with upfront surgery^32^ and predominance of hormone-responsive molecular profiles that respond poorly to neoadjuvant therapy, recent data from the I-SPY2 clinical trial showed that gene expression assays might assist in identifying patients with lobular breast cancer who are most likely to experience a nodal pCR and can safely forego ALND.^33^

Although comprehensive axillary management continues to play an important role in our breast cancer treatment armamentarium,^34^ ALND and RNI increase the risk of lymphedema, sensory nerve injury, functional deficits (e.g., reduced shoulder and arm mobility), and chronic pain.^35^ These treatment-related adverse events affect quality of life, are a major source of disability, and can contribute to financial hardship.^36–38^ Vulnerability to financial hardship is thought to be especially pronounced among certain populations, including young adult patients for whom the transition to independence is associated with a period of fiscal instability.^39^ Our finding that young adults are also more likely to receive therapies that increase the risk of costly complications emphasizes the role of tailored therapies in avoiding unnecessary treatments and mitigating financial toxicity. Given evidence that patients with limited nodal involvement may be appropriate candidates for SLNB alone with or without RNI,^6^^,^^40^ strategies to promote value-based care will allow providers to minimize the morbidity of invasive procedures and address key survivorship issues.

Despite the NCDB serving as a robust resource for analyzing treatment patterns in the USA, several limitations are worth noting. The lack of granular data to distinguish the impact of treatment details and the variability in institutional practices with respect to performing completion ALND or recommending RNI may affect the accuracy of our results. As the NCDB primarily includes facilities with Commission on Cancer accreditation,^41^ care delivered in community or rural settings may be underrepresented and impact generalizability to the broader population of patients with breast cancer. Although patients receiving neoadjuvant systemic therapy were a minority of those included in this study, the opportunity for de-escalation of axillary management among this cohort is difficult to assess; even among those who converted to pN0, clinical circumstances such as extent of pretreatment disease burden may have warranted ALND based on existing evidence. Although this study focuses on comprehensive axillary management as a risk factor for treatment-related adverse events, including BCRL, because the NCDB does not track such outcomes, further investigations are necessary to understand whether these procedures correlate with age-based differences in complications.

## Conclusion

In this study, ALND with RNI was more common in younger women. Histopathologic factors such as lobular disease and HR+ disease were more likely to require comprehensive axillary management. These data highlight an opportunity for further studies targeting management in younger cohorts to limit overtreatment.

## Data Availability

The data that support the findings of this study are derived from the NCDB. The NCDB is project supported by the Commission on Cancer (CoC). Access to NCDB data is restricted and requires approval. Researchers interested in obtaining NCDB data must submit a formal request through the NCDB Participant User File (PUF) application process.
